# Correlation between myelin basic protein levels in cerebrospinal fluid and motor speed in patients with schizophrenia

**DOI:** 10.1002/npr2.12471

**Published:** 2024-08-12

**Authors:** Takako Enokida, Kotaro Hattori, Miho Ota, Megumi Tatsumi, Shinsuke Hidese, Noriko Sato, Mikio Hoshino, Hiroshi Kunugi

**Affiliations:** ^1^ Department of Bioresources, Medical Genome Center National Center of Neurology and Psychiatry Tokyo Japan; ^2^ Department of NCNP Brain Function and Pathology, Graduate School of Medical and Dental Sciences Tokyo Medical and Dental University Tokyo Japan; ^3^ Department of Mental Disorder Research, National Institute of Neuroscience National Center of Neurology and Psychiatry Tokyo Japan; ^4^ Department of Radiology National Center Hospital of Neurology and Psychiatry Tokyo Japan; ^5^ Department of Neuropsychiatry University of Tsukuba Tsukuba Japan; ^6^ Department of Psychiatry Teikyo University School of Medicine Tokyo Japan; ^7^ Department of Biochemistry and Cellular Biology, National Institute of Neuroscience National Center of Neurology and Psychiatry Tokyo Japan

**Keywords:** cerebrospinal fluid, demyelination, motor speed, myelin basic protein, schizophrenia

## Abstract

Alterations in the white matter have been implicated in schizophrenia. Myelin basic protein (MBP), a component of the myelin sheath, in the cerebrospinal fluid (CSF) has been suggested as a biomarker for white matter damage in demyelinating diseases. This prompted us to examine the CSF‐MBP levels in patients with schizophrenia. We analyzed the CSF‐MBP levels in 152 patients with schizophrenia and 117 age‐ and sex‐matched controls. A significant positive correlation between age and CSF‐MBP levels was observed both in the patients (*p* < 0.001) and controls (*p* = 0.014). No significant difference was observed in the CSF‐MBP levels between the two groups. However, among a subsample of the patients (*N* = 32), a significantly negative correlation was observed between CSF‐MBP and age‐adjusted motor speed score of the brief assessment of cognition in schizophrenia (ρ = −0.59, *p* < 0.001). Further, among patients who underwent diffusional magnetic resonance imaging of the brain (*N* = 27), the CSF‐MBP levels showed a significantly negative correlation with the mean kurtosis value in the right temporo‐parietal region (*p* < 0.001). Our results suggest that the CSF‐MBP level has limited utility as a diagnostic marker; however, higher CSF‐MBP levels are associated with poorer motor speed, which may be associated with regional white matter damage in the brain in patients with schizophrenia.

## Abstract 2

Myelin basic protein levels in cerebrospinal fluid (CSF‐MBP) can potentially serve as a marker for aging‐related white matter damage. CSF‐MBP levels also correlated with motor speed scores in a cognitive evaluation in patients with schizophrenia.

## Graphical abstract

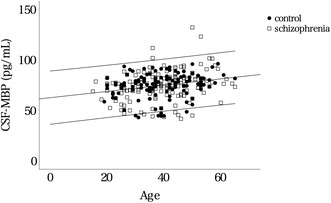

## 1 INTRODUCTION

Schizophrenia is a serious chronic mental disorder affecting approximately 1% of the population worldwide^1^ and comprises a complex set of positive, negative, and cognitive symptoms.^2^ However, the efficacy of the currently used antipsychotics is limited. Thus, an urgent need exists to advance our knowledge of its pathophysiology and identify biomarkers to develop objective diagnostic tests and precise therapeutics for subsets of the illness.^3^

Imaging studies^4^, ^5^, ^6^, ^7^, ^8^ and post‐mortem brain analyses^9^, ^10^, ^11^, ^12^ have consistently reported reduction in cerebral white matter in major psychiatric disorders, including mood disorders and schizophrenia. Additionally, recent meta‐ and mega‐analyses of imaging studies have reported white matter microstructural alterations in schizophrenia.^13^, ^14^, ^15^, ^16^ Thus, demyelination is possibly involved, at least in some brain regions, in patients with schizophrenia.

Myelin basic protein (MBP) is a component of the myelin sheath, and its elevated levels in the cerebrospinal fluid (CSF) correlate with white matter damage in multiple sclerosis (MS),^17^, ^18^ traumatic brain injury,^19^ cerebrovascular diseases, and some chronic progressive disorders, such as neurometabolic disorders.^20^ The elevated CSF‐MBP levels in MS are believed to be attributed to myelin sheath destruction due to immunological disturbances.^21^ Interestingly, certain subgroups of patients with schizophrenia exhibit blood–brain barrier impairment and elevated inflammatory marker levels.^22^, ^23^, ^24^

In this study, we investigated potential alterations in the CSF‐MBP levels in patients with schizophrenia in comparison with those of healthy controls. We also examined the possible association of the CSF‐MBP levels with clinical variables, including cognitive functions. Additionally, we analyzed the correlation between the CSF‐MBP levels and the microstructure of the whole brain in patients with schizophrenia using diffusional magnetic resonance imaging (dMRI).

## 2 METHODS

### 2.1 Participants

The recruitment and evaluation of participants, as well as the collection, storage, and preparation of CSF and blood samples, were supported by the National Center of Neurology and Psychiatry (NCNP) Biobank, Tokyo, Japan, a member of the National Center Biobank Network.^25^ Patients were recruited at the NCNP Hospital in Tokyo, Japan, or via website announcements. Control participants were recruited through advertisements in a free local magazine and our website announcements. Participants with a history of central nervous system diseases, severe head injuries, or substance abuse were excluded.

All participants underwent structured interviews using the Japanese version of the Mini‐International Neuropsychiatric Interview (M.I.N.I.),^26^, ^27^ conducted by trained psychologists or psychiatrists. A consensus diagnosis was made according to the Diagnostic and Statistical Manual of Mental Disorders, Fourth Edition criteria,^28^ based on the M.I.N.I., additional unstructured interviews, and information from medical records, if available, for participants with schizophrenia. Most patients were chronic cases under antipsychotic medication. Medication status at the time of lumbar puncture was recorded. Daily doses of antipsychotics were converted to chlorpromazine (CP)‐equivalent doses, according to published guidelines.^29^ Schizophrenic symptoms were assessed using the Japanese version of the Positive and Negative Syndrome Scale (PANSS).^30^ This study used cerebrospinal fluid samples which had already been collected in NCNP biobank. Among the participants who provided CSF samples, only a limited number of patients underwent MRI or cognitive functional evaluation. We used all available clinical data connected with the cerebrospinal fluid samples.

This study was conducted in accordance with the tenets of the Declaration of Helsinki^31^ and approved by the Ethics Committee of the NCNP, Japan (A2012‐091, A2019‐092). All participants provided written informed consent.

### 2.2 Assessment of cognitive functions

Among the total 152 patients with schizophrenia, 32 (15 female patients; mean age ± standard deviation [SD] = 40.0 ± 9.8 years) were assessed for cognitive functions using the Japanese version of the brief assessment of cognition in schizophrenia (BACS‐J).^32^, ^33^ The BACS assesses six categories of cognitive functions, that is, verbal memory, working memory, motor speed, verbal fluency, attention, and executive function, which yield an overall composite score. The raw scores were converted to z‐scores by referring to the mean and SD of the BACS‐J, stratified by sex and age.^34^

### 2.3 CSF sample collection

CSF samples were obtained through lumbar puncture, as previously described.^35^ Selected samples were thawed once and stored in polypropylene tubes until further use.

### 2.4 Measurement of MBP in the CSF

The CSF‐MBP levels were measured using an enzyme‐linked immunosorbent assay (ELISA) kit for human MBP (DY4228‐05; R&D Systems, Minneapolis, MN, USA), according to the manufacturer's instructions. Initially, the measurement was verified using the pooled CSF samples. The coefficient of variation was <7%. The CSF sample used in the measurement was 60 μL and was not diluted. ELISA measurements were performed in a single‐blinded manner by a single researcher (T.E.). Between‐plate normalization was performed using overlapping samples from the plates. Between‐batch normalization was performed by the z‐score transformation of the control samples.

### 2.5 MRI data acquisition and processing

Among the 152 patients with schizophrenia, 27 (14 female patients; 35.6 ± 11.2 years) underwent MRI of the brain within 3 months before or after lumbar puncture. MRI was performed on a 3‐T MR system (Achieva, Philips Medical Systems, Best, The Netherlands). High spatial resolution, three‐dimensional (3D) T1‐weighted images were used for morphometric study. The 3D T1‐weighted images were acquired in the sagittal plane (repetition time [TR]/echo time [TE], 7.18/3.46; flip angle, 10°; effective section thickness, 0.6 mm; slab thickness, 180 mm; matrix, 384 × 384; field of view [FOV], 261 × 261 mm; number of signals acquired, 1), yielding 300 contiguous slices through the brain. For diffusion imaging, a multishell protocol was acquired along 16 non‐collinear directions at two b‐values (1000, 2000 s/mm^2^), and one image was acquired without using any diffusion gradient. To increase the signal‐to‐noise ratio, the high b‐value images (b = 2000 s/mm^2^) were acquired with number of excitations = 2. The acquisition parameters were as follows: TE/TR = 87/6767 ms, FOV = 240 × 240, matrix = 96 × 96, and voxel dimension of 2.5 × 2.5 × 2.5 mm^3^, with 60 slices acquired in ascending order.

### 2.6 Postprocessing of the dMRI data

The data underwent denoising and Gibbs ringing correction by MRtrix3.^36^, ^37^ Susceptibility distortions were corrected using the Synb0‐DisCo approach,^38^ and eddy current‐induced distortions of diffusion images were corrected using FMRIB's Software Library version 6.0.^39^ Mean kurtosis (MK) maps were calculated using the Diffusional Kurtosis Estimator.^40^ To exclude the extraparenchymal noise, we masked the MK maps with the binary mask image made by the co‐registered and resliced individual 3D‐T1 image.

### 2.7 MRI data analysis

To evaluate the MK value of the whole‐brain by voxel‐based analysis, these images were normalized using the template “EPI.nii,” which is the standard image for SPM8, as a reference. Finally, each image was smoothed using a 5‐mm full‐width at half‐maximum Gaussian kernel.

### 2.8 Statistical analysis

The Kolmogorov–Smirnov test was used to assess the normal distribution. Sex differences were compared using the chi‐square test. Age was compared using the unpaired *t*‐test. MBP was compared using the analysis of covariance (ANCOVA), controlling for age and sex. The associations between CSF‐MBP levels and age were evaluated using Spearman's correlation test. Partial correlation analysis with age and sex as covariates was used to analyze correlation between CSF‐MBP levels and clinical indicators (PANSS and BACS). Statistical significance was set at *p* < 0.05. All statistical analyses were conducted using IBM SPSS (version 29; SPSS Software, Inc., Chicago, IL, USA).

For MRI data analyses, the correlation between the MK value and CSF‐MBP levels was evaluated by SPM8, using sex and age as nuisance variables. The significance level was set at a seed level of *p* < 0.001 and a cluster level of *p* < 0.05. All *p* values, including those for the demographic and correlation analyses, were uncorrected for multiple testing.

## 3 RESULTS

**FIGURE 1:**
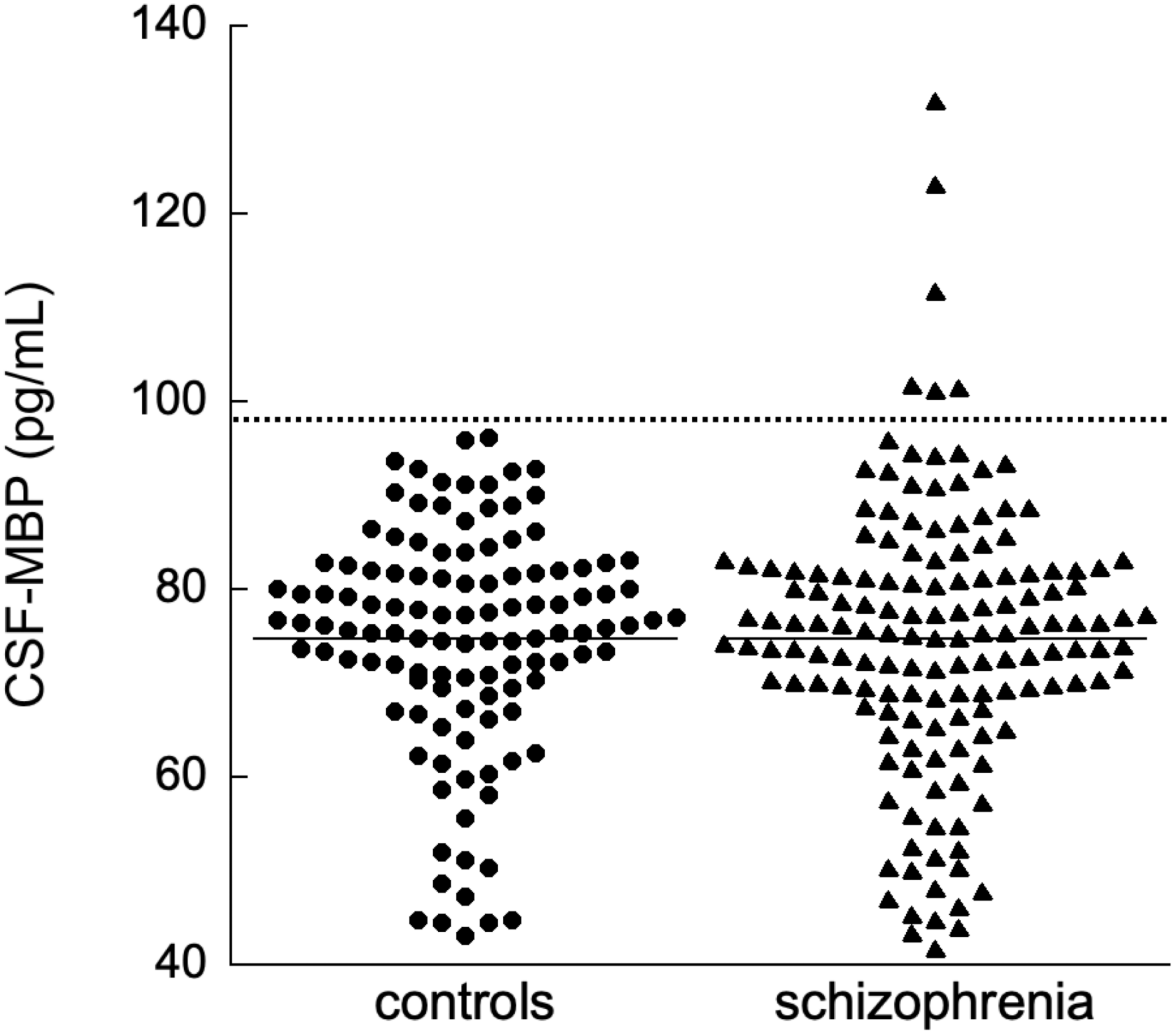
Dot plots with the MBP levels in the CSF samples of controls and patients with schizophrenia. The horizontal bar represents the mean values of each group. The dotted line represents the upper limit of normal, which is the mean of the controls plus two times the standard deviation (=98.6 pg/mL). CSF, Cerebrospinal fluid; MBP, Myelin basic protein.

**FIGURE 2:**
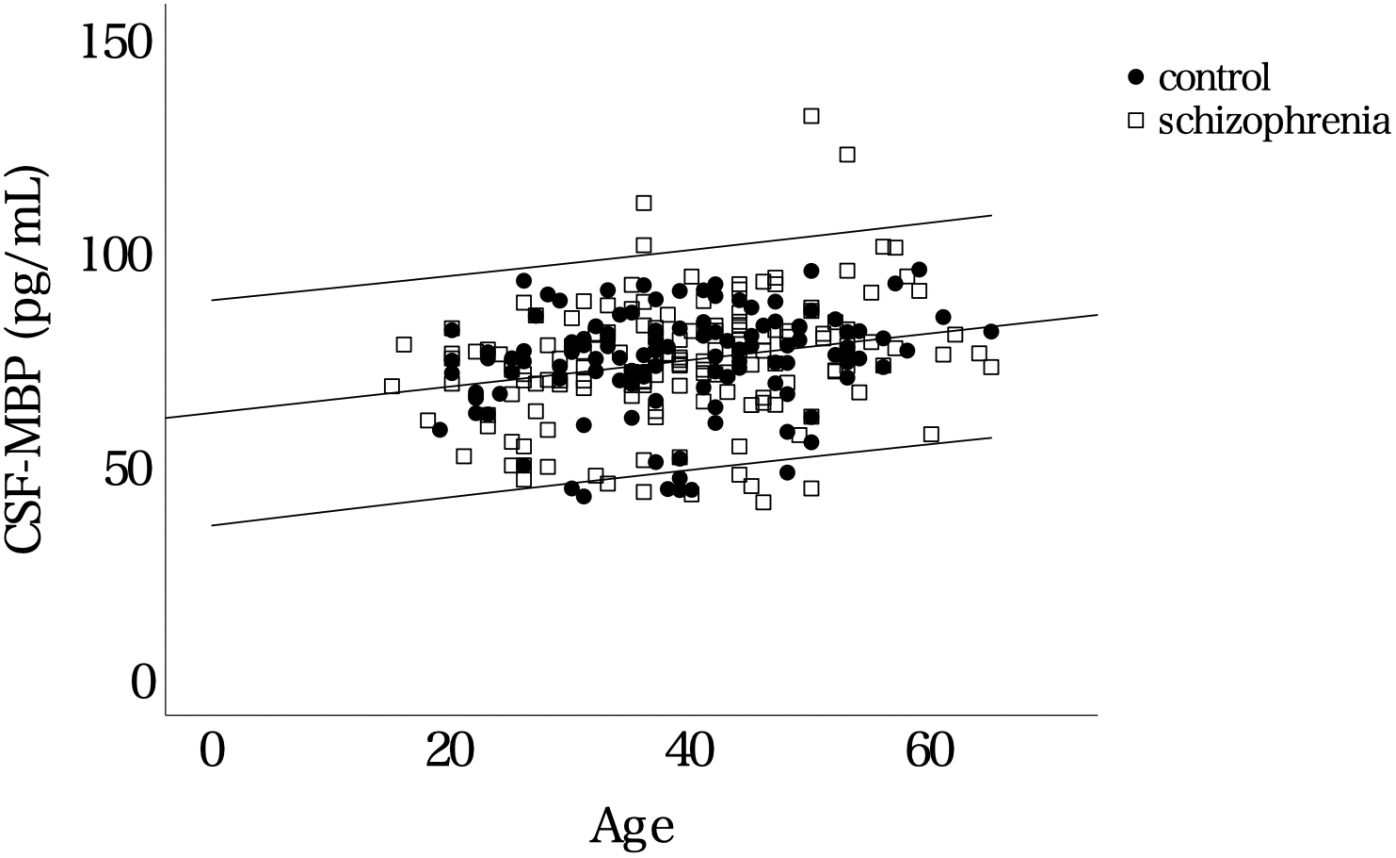
Scatter plots with age on the *x*‐axis and the CSF‐MBP levels on the *y*‐axis. The maximum and minimum ages of the participants were 65 and 15, respectively. The middle line is the regression line, and the upper and lower dashed lines represent 95% CI. CI, Confidence interval; CSF, Cerebrospinal fluid; MBP, Myelin basic protein.

We measured the CSF‐MBP levels in 152 patients with schizophrenia and 117 age‐ and sex‐matched controls. Table 1 summarizes the demographics and clinical data of the participants. The distributions of the CSF‐MBP levels in the two groups are shown in Figure 1. The Kolmogorov–Smirnov test indicated a non‐normal distribution of the CSF‐MBP levels in the schizophrenia and control groups (*p* = 0.003, *p* = 0.001, respectively). Age showed a significantly positive correlation with CSF‐MBP levels both in the schizophrenia (Spearman's ρ = 0.28, *p* < 0.001) and control (ρ = 0.23, *p* = 0.014) groups (Figure 2). No significant difference in the CSF‐MBP levels was observed between the schizophrenia and control groups (*p* = 0.99, ANCOVA, controlling for age and sex). Additionally, no significant difference in the CSF‐MBP levels was observed between male and female patients in either diagnostic group (*p* > 0.1 for both groups).

**TABLE 1:** Demographics and clinical data of the participants.

Diagnosis	Controls	Patients	*p* value (test, degree of freedom [df])
Number	117	152	
Age (mean ± SD) (Min–Max)	38.9 ± 10.5 (19–65)	39.1 ± 10.9 (15–65)	*p* = 0.89 (*T*‐test, df = 1)
Sex, male (%)	70 (59.8%)	91 (59.9%)	*p* = 0.99 (χ^2^ test, df = 1)
CP‐equivalent	–	526.4 (253–888)	–
CSF‐MBP median (interquartile range) (pg/mL)	76.1 (69.8–82.1)	75.3 (68.5–82.0)	*p* = 0.99 (ANCOVA)

Abbreviations: CP‐equivalent, chlorpromazine‐equivalent; CSF‐MBP, cerebrospinal fluid‐myelin basic protein.

**FIGURE 3:**
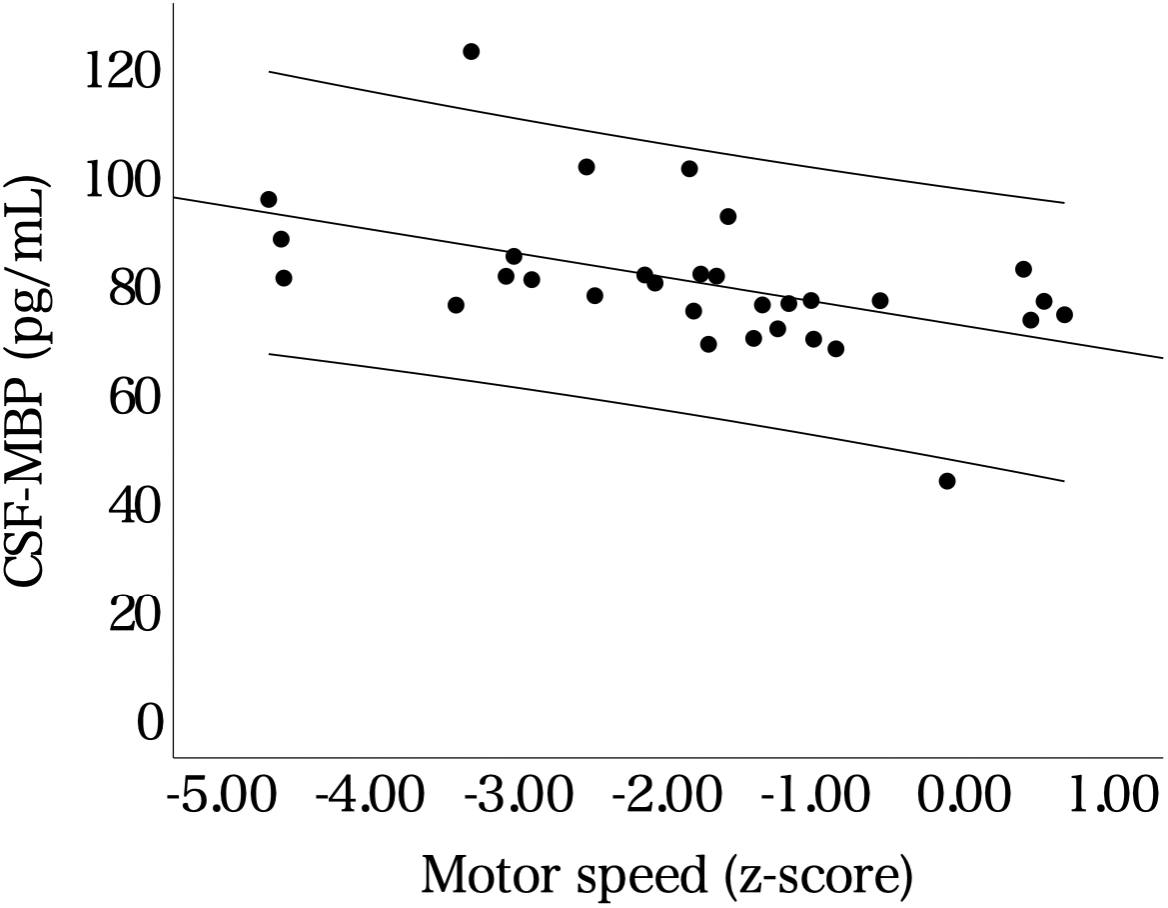
Scatter plots of the data of the patients with schizophrenia with the CSF‐MBP levels on the *x*‐axis and the motor speed scores of BACS on the *y*‐axis. The middle line is the regression line, and the upper and lower dashed lines represent 95% CI. The maximum and minimum ages of the participants were 56 and 20, respectively. BACS, The brief assessment of cognition in schizophrenia; CI, Confidence interval; CSF, Cerebrospinal fluid; MBP, Myelin basic protein.

**FIGURE 4:**
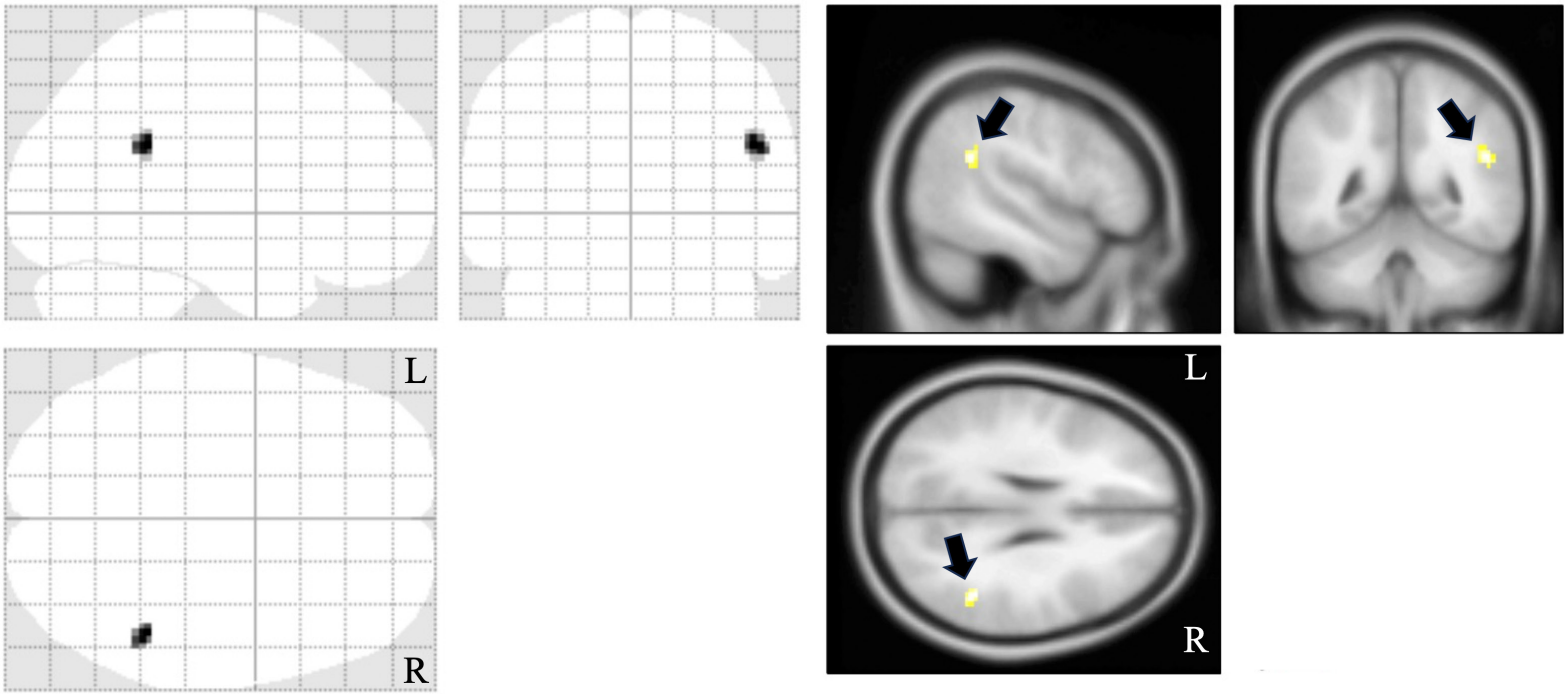
Relationships between the MK value and CSF‐MBP level in the patients with schizophrenia. A significant negative correlation was observed between the MK value and CSF‐MBP level in the right temporo‐parietal region (indicated by arrows in the three right images). The maximum and minimum ages of the participants were 57 and 20, respectively. The background image is the “avg152T1” image, regarded as one of the anatomically standardized images in the SPM8. “R” is right, and “L” is left. CSF, Cerebrospinal fluid; MBP, Myelin basic protein; MK, Mean kurtosis.

Furthermore, no significant correlation was observed between the CSF‐MBP levels and PANSS score (partial correlation = −0.05, *p* = 0.62) or CP‐equivalent values (partial correlation = 0.2, *p* = 0.076). In a subsample of the patients (*n* = 32) assessed for cognitive functions using BACS, we observed no significant correlation between the CSF‐MBP levels and composite scores of BACS (Table 2). However, when each cognitive function was examined, we detected a highly significant negative correlation between the CSF‐MBP levels and motor speed scores (partial correlation = −0.49; *p* = 0.006) (Table 2, Figure 3). Finally, analysis of the brain MRI data of some patients (*n* = 27) revealed a significantly negative correlation between the CSF‐MBP levels and MK values in the right temporo‐parietal region (*p* < 0.001 [uncorrected]) (Figure 4).

**TABLE 2:** Correlation analysis of the CSF‐MBP levels and BACS scores.

	Patients with schizophrenia (*n* = 32)			
	Median raw score (interquartile range)	Median z‐score (interquartile range)	*p* Value	Partial correlation
Verbal memory	36.5 (25.5 to 45.8)	−1.3 (−2.2 to −0.25)	*p* = 0.83	−0.04
Working memory	18.0 (15.0 to 21.0)	−0.78 (−1.4 to 0.091)	*p* = 0.24	−0.22
Motor speed	68.0 (56.0 to 78.0)	−1.7 (−2.9 to −1.0)	*p* = 0.006*	−0.49
Verbal fluency	21.0 (16.3 to 23.8)	−0.22 (−1.6 to 0.67)	*p* = 0.35	−0.18
Attention	24.5 (13.8 to 29.8)	−1.3 (−2.8 to −0.91)	*p* = 0.19	−0.25
Executive function	52.0 (38.5 to 57.8)	−0.43 (−1.5 to 0.064)	*p* = 0.17	−0.26
Composite score	17.0 (15.0 to 18.0)	−1.8 (−2.7 to −1.1)	*p* = 0.99	0.0

Abbreviations: BACS, Brief assessment of cognition in schizophrenia; MBP, Myelin basic protein.

*indicates significant values *p* < 0.05

## 4 DISCUSSION

In the present study, we measured the CSF‐MBP levels in patients with schizophrenia and age‐ and sex‐matched controls, revealing no significant differences between the two groups. However, a positive correlation was observed between the CSF‐MBP levels and age. Interestingly, a negative correlation was observed between the CSF‐MBP levels and motor speed scores of BACS. Further, dMRI analysis indicated a negative correlation between the CSF‐MBP levels and MK values in the right temporo‐parietal region, suggesting structural abnormalities in the white matter.

Previous studies have indicated white matter abnormalities in patients with schizophrenia. Post‐mortem studies have reported decreased numbers of oligodendrocytes in the prefrontal cortex,^9^ superior frontal gyrus,^10^ and anterior hippocampus^11^ in schizophrenia. Imaging studies have suggested a reduction in white matter volume in schizophrenia in specific regions, including the left fronto‐occipital fasciculus,^4^ corpus callosum,^41^ and superior longitudinal fasciculus (SLF).^42^ Recent large multicenter studies using diffusion‐weighted imaging, diffusion‐tensor imaging, and meta‐analyses have suggested correlations between schizophrenia and white matter microstructural alterations.^13^, ^14^, ^15^, ^43^

MBP, a major component of the myelin sheath, has been suggested to be an indicator of white matter disorders in clinical practice. Two previous studies measured the CSF‐MBP levels in patients with schizophrenia. Steiner et al.^44^ examined the CSF‐MBP levels in 12 patients with first‐episode schizophrenia and 17 controls and reported no significant differences in the mean levels between the two groups. Similarly, Li et al.^45^ analyzed the CSF‐MBP levels in 33 patients with first‐episode schizophrenia and nine controls and observed no differences between the groups. Although most patients in this study were in the chronic phase of schizophrenia, we obtained data from a much larger number of patients and observed no significant difference in the CSF‐MBP levels between the patient and control groups. In summary, no evidence suggests an overall elevation of CSF‐MBP levels in patients with schizophrenia, indicating its limited utility as a diagnostic marker for schizophrenia.

Figure 1 illustrates that six patients (3.9%) exhibited abnormally high MBP levels (i.e., higher than mean + 2 SD in controls). However, we could not identify any specific clinical features associated with these patients (data not shown). Since the number of patients was small, further studies are required to determine whether such patients with abnormally high CSF‐MBP levels should be classified as having a subtype of schizophrenia.

We found a positive correlation between the CSF‐MBP levels and age, consistent with the findings which showed an age‐dependent increase in the CSF‐MBP levels.^46^ These observations are plausible because white matter is affected by age,^47^ and white matter reduction in patients with schizophrenia is suggested to accelerate with age.^48^, ^49^ Myelin fragmentation has been reported to increase with age, and aging myelin sheaths are subsequently removed by microglia, leading to lysosomal storage and contributing to microglial senescence.^50^ One reason for the increase in MBP may be the increase of the destruction of the myelin sheath. Another possibility is decreased clearance rates of MBP in CSF. The glymphatic system is a recently discovered clearance mechanism in the central nervous system that shows an age‐related decline in activity, potentially leading to protein accumulation.^51^ The increase in CSF‐MBP with age may also reflect decreased clearance by the glymphatic system. Overall, the underlying cause of elevated CSF‐MBP could be attributed to both increased age‐related white matter degradation and potential clearance reduction.

Interestingly, we observed a negative correlation between the CSF‐MBP levels and motor speed scores of BACS in patients with schizophrenia. To our knowledge, this is the first study to reveal such a correlation. Myelin integrity is thought to be essential for high‐frequency action potential bursts in the brain, which is required for maximum motor speed.^52^ Previous studies on patients with MS^53^ or healthy older adults (>60 years and free of dementia)^54^ have reported correlations between decreased fractional anisotropy (FA) in normal‐appearing white matter and reduced motor speed. These reports indicate that even in the absence of gross structural abnormalities, the myelin breakdown is associated with decreased motor speed. Further, brain aging has been associated with cognitive impairment, including motor speed, and myelin breakdown may be a contributing factor.^52^, ^55^ In summary, CSF‐MBP level can potentially serve as a marker for aging and aging‐related deterioration of the myelin sheath responsible for motor speed. Further studies are warranted to examine whether the relationship between the CSF‐MBP level and motor speed exists in individuals without schizophrenia.

We found a significantly negative correlation between the CSF‐MBP levels and MK values in the right temporo‐parietal region in patients with schizophrenia. The SLF have tracts run through this region. SLF is a set of white matter bundles connects various brain regions, including the frontal, parietal, and temporal lobes. Previous studies have reported reductions in the SLF in patients with schizophrenia,^16^, ^56^ implicating its role in cognitive impairments in schizophrenia. Further, a correlation between the motor speed score of BACS and the FA values in white matter bundles was reported, including SLF.^57^ There were also likely subcortical U fibers connecting between cortical region and adjacent gyri. Thus, it is possible that deterioration in the white matter in the right temporo‐parietal region is responsible for impaired motor speed in schizophrenia and that MBP is a potentially important target molecule for drug discovery for treating cognitive impairment.

Most of the patients in this study had chronic disease and were under antipsychotic medication. Therefore, it is still unclear whether the CSF‐MBP levels were altered in the acute phase of the illness. Concerning medication, we found no significant correlation between the CP‐equivalent values and CSF‐MBP levels, suggesting that the possible effect of antipsychotic medication on CSF‐MBP might be minimal.

We measured the CSF‐MBP levels in a relatively large number of patients with schizophrenia and age‐ and sex‐matched controls. Furthermore, we examined the possible correlation of the CSF‐MBP levels with cognitive functions and brain dMRI in some patients. Our results suggest that the CSF‐MBP level may not serve as a diagnostic marker; however, higher CSF‐MBP levels were associated with poorer motor speed function, which might be related to regional white matter damage in the brain of patients with schizophrenia.

## AUTHOR CONTRIBUTIONS

Conceptualization, T.E., K.H. and H.K.; methodology, M.O. and M.T.; formal analysis, T.E. and K.H.; investigation, T.E.; resources, T.E., K.H., M.O., M.T., S.H., N.S.; writing—original draft preparation, T.E. and K.H.; writing—review and editing, H.K.; supervision, M.H. and K.H.; funding acquisition, T.E. and K.H. All authors have read and agreed to the published version of the manuscript.

## FUNDING INFORMATION

This study supported by Intramural Research Grant (2‐Y1) for Neurological and Psychiatric Disorders of NCNP (T.E.), and Intramural Research Grant (3‐1) for Neurological and Psychiatric Disorders of NCNP (K.H.). They played no role in the study design; the collection, analysis and interpretation of data, in the writing of the report; and in the decision to submit the paper for publication.

## CONFLICT OF INTEREST STATEMENT

The authors declare no conflict of interest.

## ETHICS STATEMENT

Approval of the Research Protocol by an Institutional Reviewer Board: This study was approved by the Ethics Committee of the NCNP, Japan (A2012‐091, A2019‐092).

Informed Consent: All participants provided written informed consent.

Registry and Registration No. of the Study: N/A.

Animal Studies: N/A.

## Supporting information


Data S1:

## Data Availability

The data that supports the findings of this study are available in the supplementary material of this article.
